# Molecular characterization and functional insights of secondary hair follicles across the cashmere growth cycle

**DOI:** 10.1186/s12864-026-12885-7

**Published:** 2026-04-25

**Authors:** Yanlei Liu, Minglin Wang, Yiping Wei, Xinyue Liang, Hongji Yu, Hongxia Li, Bohan Liu, Junpeng Zhang, Xinghui Zhang, Wenlin Bai, Songyang Shang, Huiling Xue

**Affiliations:** 1 College of Animal Science and Veterinary Medicine, Shenyang Agricultural University (SYAU), Shenyang, 110866 China; 2 Liaoning Provincial Agricultural and Rural Development Service Center, Shenyang, 110001 China

**Keywords:** Cashmere, Histological observation, KRT and KRTAP, Secondary hair follicle, Transcriptome

## Abstract

**Background:**

Cashmere production relies on the circannual regeneration of secondary hair follicles (SHFs), yet the precise timing, morphology, and molecular regulation of the SHF cycle in cashmere goats remain incompletely defined.

**Results:**

In the present study, we integrate a year-long longitudinal histological analysis with SHF-specific transcriptomic profiling to establish a comprehensive framework of SHF dynamics in Liaoning cashmere goats. High-resolution morphological observation reveals the full-cycle alterations in SHF architecture. Microscopically isolated SHFs yielded expression profiles that accurately defined follicular states and unequivocally distinguished anagen, catagen, and telogen. Across the annual cycle, the telogen-to-early anagen transition emerges as the dominant regulatory shift, accounting for the majority of differentially expressed genes. These signatures function in coordinated opposing patterns, converging on pathways involved in stem cell priming, follicle reactivation, metabolism, and tissue remodeling. In contrast, anagen is characterized by upregulation of genes governing translation, RNA processing, oxidative phosphorylation, and thermogenesis, reflecting the elevated biosynthetic demand during fiber elongation. We further delineate phase-specific regulation of KRT and KRTAP gene families, revealing distinct temporal regulation with direct implications for fiber quality. Co-expression network analysis identifies four major gene modules aligned with specific phases, including a large telogen-enriched module that underscores the transcriptional activity of this traditionally quiescent state.

**Conclusions:**

In summary, this study delivers a refined, biologically validated model of the annual SHF cycle, offering new mechanistic insight into the molecular programs that orchestrate seasonal cashmere growth and shedding.

**Supplementary Information:**

The online version contains supplementary material available at 10.1186/s12864-026-12885-7.

## Background

Cashmere is a fine, soft fiber distinguished by its resilience, superior thermal insulation, and considerable economic value. In cashmere goats, these fibers are produced exclusively by secondary hair follicles (SHFs), whereas the coarser protective hairs arise from primary hair follicles (PHFs) in the double-coated skin [1]. SHFs are smaller than PHFs, typically arranged in clustered units surrounding a central PHF, and exhibit distinct morphological and developmental characteristics [2, 3]. Similar to other mammalian hair follicles, SHFs regenerate cyclically, transitioning through quiescent (telogen), growth (anagen), and regression (catagen) phases [4, 5]. However, unlike the asynchronous growth observed in goat wool or horse mane, SHFs undergo a strikingly synchronized annual cycle, yet the mechanisms that coordinate their temporal transitions remain incompletely understood. Elucidating the dynamics and molecular regulation of this synchronized cycle is essential for improving fiber yield and quality. Here, we characterize the annual dynamic program of SHFs and uncover key regulatory signatures that orchestrate the seasonal initiation, elongation, and shedding of cashmere fibers.

Previous studies mostly examined whole goat skin, leaving SHF-specific molecular characteristics less understood. Goat skin comprises multiple cell types and both SHFs and PHFs, which exhibit distinct responses to regulatory cues. For example, PHFs are absent in *Eda*-mutant Tabby mice, whereas SHFs still form in normal numbers, albeit with aberrantly shorter, thinner, and straighter hairs [6–8]. The Wnt antagonist *Dkk4* selectively disrupts SHF development, with primary hairs remaining normal in *Dkk4* transgenic mice, whereas secondary hairs are severely malformed [9]. Sonic Hedgehog (*Shh*) is correlated with the formation of both hair types, but in different ways with differential consequences [10]. Melatonin increases SHF initiation and maturation with minimal impact on PHF [2]. These observations indicate that SHFs and PHFs are governed by partially distinct molecular mechanisms, underscoring the importance of analyzing SHFs in isolation.

The keratin (KRT) and keratin-associated protein (KRTAP) families are fundamental to the structure, function, and quality of hair and wool fibers, including cashmere. Keratins are intermediate filament (IF) proteins that provide structural integrity and resilience to epithelial and follicular cells, while KRTAPs are small matrix proteins rich in cysteine, glycine, and tyrosine, which bind keratin filaments to form the hair shaft’s microfibrils. These proteins are crucial in defining fiber diameter, curvature, tensile strength, and thus directly influence hair quality [11–14]. Expression of individual KRT and KRTAP genes is tightly regulated in the hair follicle. In cashmere goats, sheep, and humans, specific *KRTs* (e.g., *KRT31*, *KRT33A*, *KRT35*, *KRT85*, *KRT71*–*KRT74*) and *KRTAPs* (such as *KRTAP7-1*, *KRTAP8-1* and *KRTAP11-1*) exhibit spatially restricted expression in the inner root sheath (IRS) and hair cortex—regions responsible for fiber elongation and structure formation [15–23]. Studies in cashmere goats consistently implicate *Krt26*, *Krt28*, *Krt39*, *Krtap8*, and *Krtap9-2*, and related genes as key contributors to fiber fineness, diameter, and follicle cycling [11–13, 24]. Comparable associations in sheep and rabbits highlight both conserved and species-specific functional roles within these gene families [25, 26]. Beyond their structural roles, these proteins mediate signal transduction in growth and wound repair [27–29]. Understanding how KRT and KRTAP genes are deployed across follicular compartments and developmental stages is therefore critical for elucidating the molecular basis of fiber formation.

In our previous work, we established an SHF-specific sampling strategy by microscopically isolating SHF clusters from back skin after removal of epidermis and connective tissue [3, 30]. RNA sequencing was then performed on these collected SHF clusters in twelve samples across six-time points—spanning anagen (May, September), catagen (November, December), and telogen (February, March)—generated a reference transcriptomic framework for SHF cycling [30]. Building on this foundation, the present study integrates histological and transcriptomic analyses of SHFs across an annual cycle. We establish a refined and systematic characterization of hair follicle dynamics, identify genes preferentially expressed in SHFs relative to mixed skin, and define phase-specific transcriptional signatures that regulate seasonal fiber growth. Together, these analyses establish a comprehensive framework for understanding the biological mechanisms that drive cashmere follicle cycling and lay the groundwork for future mechanistic and breeding-based improvements in cashmere production.

## Methods

### Experimental animals

All experimental cashmere goats were from the Liaoning Provincial Agricultural and Rural Development Service Center, Shenyang. Samples were collected from thirty-six 1.5-year-old cashmere goats across six time points (February, March, May, September, November, and December). Each time point included six goats, consisting of three males and three females. The SHFs were isolated by cutting out the epidermis and removing the connective tissues under a dissecting microscope (SMZ150, Motic, Xiamen, China) from a piece of back skin (0.5 × 0.5 cm) of each goat. The detailed experiment procedure please refer to our previous work [3, 30]. All the experimental goats were fed with cashmere goat standard feed (NY/T 2893 − 2016). All the animal studies were reviewed and approved by the Ethical Committee of Shenyang Agricultural University, China (ID no. 2021121701).

### Immunofluorescence staining of skin tissues

Dorsal skin samples were collected from the mid-dorsal region of cashmere goats. Immunofluorescence staining was performed as previously described [3]. The antibodies used for immunofluorescence included the following: KRT17 (1:300; Abcam, Cambridge, UK) and KRT14 (1:500; Abcam), followed by corresponding secondary antibodies: Alexa Fluor 488-conjugated (1:400; Invitrogen, Sydney, Australia) and Alexa Fluor 594-conjugated (1:400; Abcam), respectively. Nuclei were counterstained with DAPI.

### RNA library preparation and RNA sequencing

Total RNA was obtained from the collected SHFs by using RNAiso Plus, according to the manufacturer’s instructions (Takara, Dalian, China). Transcriptome profiling was performed by RNA-seq using the Illumina HiSeq 4000 sequencing platform at Novogene (Beijing, China). The raw RNA-seq data have been submitted to the NCBI Gene Expression Omnibus (GEO) database under the accession number GSE221472. The method for data processing is detailed in our previous publication [30].

### Sequencing analysis

The pipeline for sequence alignment and gene expression quantification was implemented as detailed in our previous publication [30]. Differentially expressed genes between the different phases were identified using the Sleuth package in R [31]. The filtered criteria used were: (1) TPM ≥ 10 in at least two samples to remove low-abundance transcripts, (2) an adjusted *p*-value < 0.05, and (3) the threshold of expression change is b ≥ 0.4 (b =|ln (fold change)|).

### Clustering and functional enrichment analysis

Hierarchical clustering was performed using the pheatmap package (v.1.0.12) in R based on the TPM data for DEGs. Enriched GO (biological process) and KEGG pathway (cellular processes, environmental information processing, and organismal systems) analyses were performed using the R module of clusterProfiler (v.4.4.4). An adjusted *p* value < 0.05 was considered statistically significant.

### Weighted gene co-expression network analysis (WGCNA)

A weighted gene co-expression network was constructed with highly expressed genes using the weighted correlation network analysis (WGCNA, v.1.70.3) package in R (v.4.0.2) [32]. A total of 5,162 genes (TPM ≥ 30 in at least two samples) were used to construct the network. Then, soft-thresholding power β values set to 8 to calculate network structures under an unsigned model. The hierarchical clustering tree algorithm was used to determine the co-expression network module. Modules with a high correlation (*R* ≥ 0.875) of characteristic genes were merged into one module.

### Quantitation of selected mRNA via RT-qPCR

The complementary DNA (cDNA) was generated by using the PrimeScript RT reagent Kit with the gDNA Eraser (Takara, Dalian, China), according to the manufacturer’s protocol. The primers used for RT-qPCR were designed with Primer (v.5.0) software and synthesized by Sangon Biotech Co., Ltd. (Shanghai, China). The RT-qPCR was conducted on the QuantStudio 3 Real-time PCR Instrument (Thermo Fisher, Waltham, MA, USA) with the SYBR Premix Ex Taq II (Takara, Dalian, China). Relative gene expression was calculated by the 2^−ΔΔCt^ method, with *Actb* (Actin beta) as the reference control. Statistical analysis was performed using SPSS software (v.15.0). Values are represented as means ± standard deviations. A significance level of 0.05 was used. All the primers used for RT-qPCR are listed in Supplementary Table S1.

### Statistical analysis

GraphPad Prism software (v.8.0.1) was used to analyze histograms with a *t*-test. A *p*-value of < 0.05 was considered statistically significant.

## Results

### Histological analysis of SHFs across the annual hair cycle

**Fig. 1:**
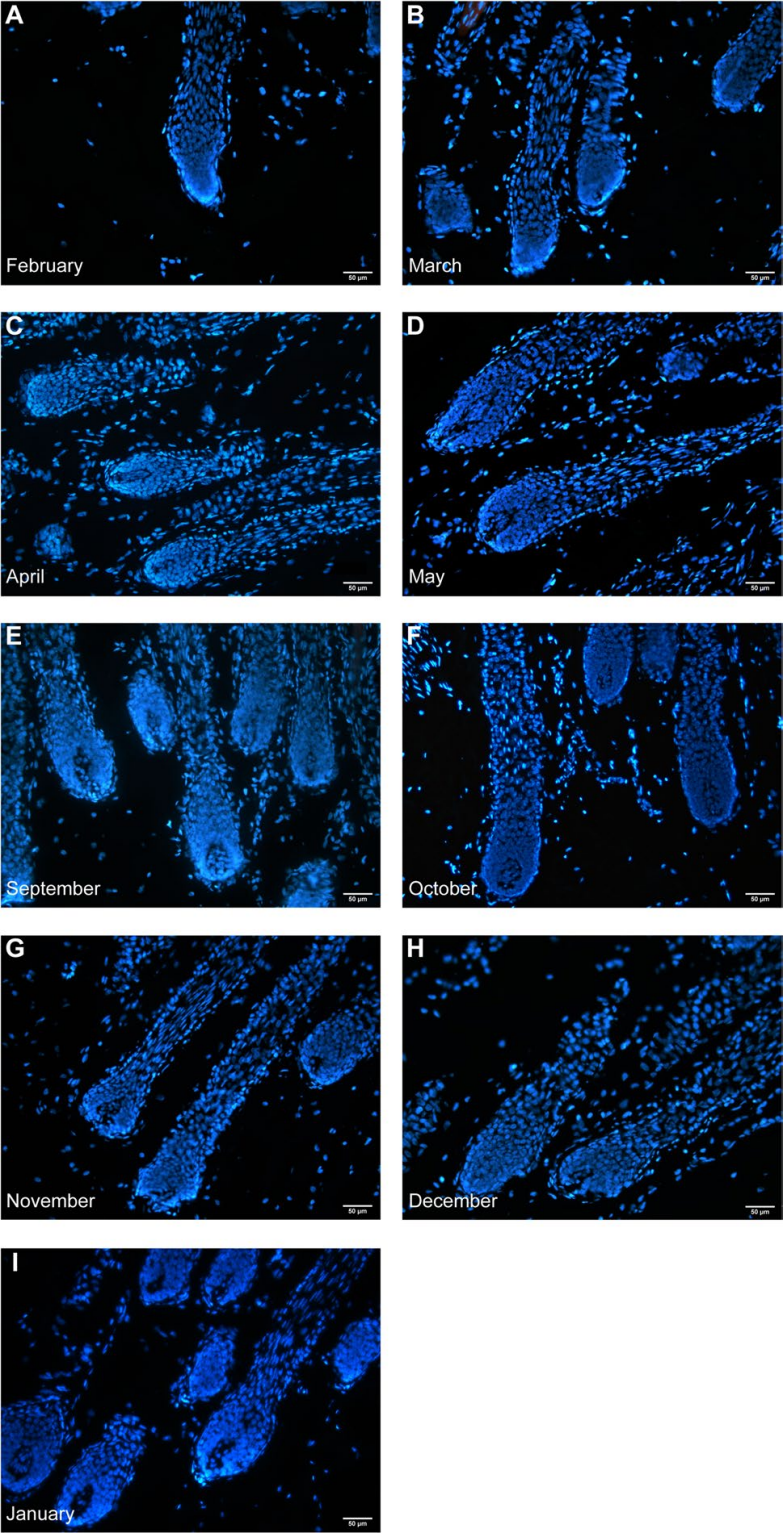
Histological observations of secondary hair follicles (SHFs) across one year. Histological alterations were observed in three phases, including telogen (**A**, **B**), early anagen (**C**, **D**), mid-anagen (**E**, **F**), and catagen (**G**, **H**, **I**). The nuclei were stained with DAPI (blue). Bar = 50 μm

To define the growth phases of SHFs in Liaoning cashmere goats, we examined histological alterations associated with distinct phases of the hair cycle over a year (Fig. 1). From February to March, SHFs were retracted to their smallest size and the dermal papilla (DP) positioned close to the bulge, consistent with telogen. During April and May, SHFs began to elongate, and the hair bulb, containing matrix cells surrounding the DP, progressively enlarged, marking the transition into early anagen. By September and October, the hair bulb appeared fully developed, a hallmark of anagen. As follicles entered catagen in November, the bulb started to shrink, losing its rounded structure, signaling the onset of the early regression. In December and January, this regression became pronounced, with marked shrinkage and upward retraction of the lower follicle. Together, these observations suggest that SHFs in Liaoning cashmere goats undergo synchronized annual hair cycle of relative quiescence from February to March (telogen), active growth from April to October (anagen), and progressive regression from November to January (catagen). This comprehensive longitudinal morphological analysis of SHFs, which enabled direct visualization of changes in hair bulb volume, structural remodeling during follicular elongation and regression, and the intermediate transitions between phases, provides a robust reference framework for subsequent studies of cashmere follicles.

### Genome-wide profiling defines SHF growth states during annual cashmere growth

**Fig. 2:**
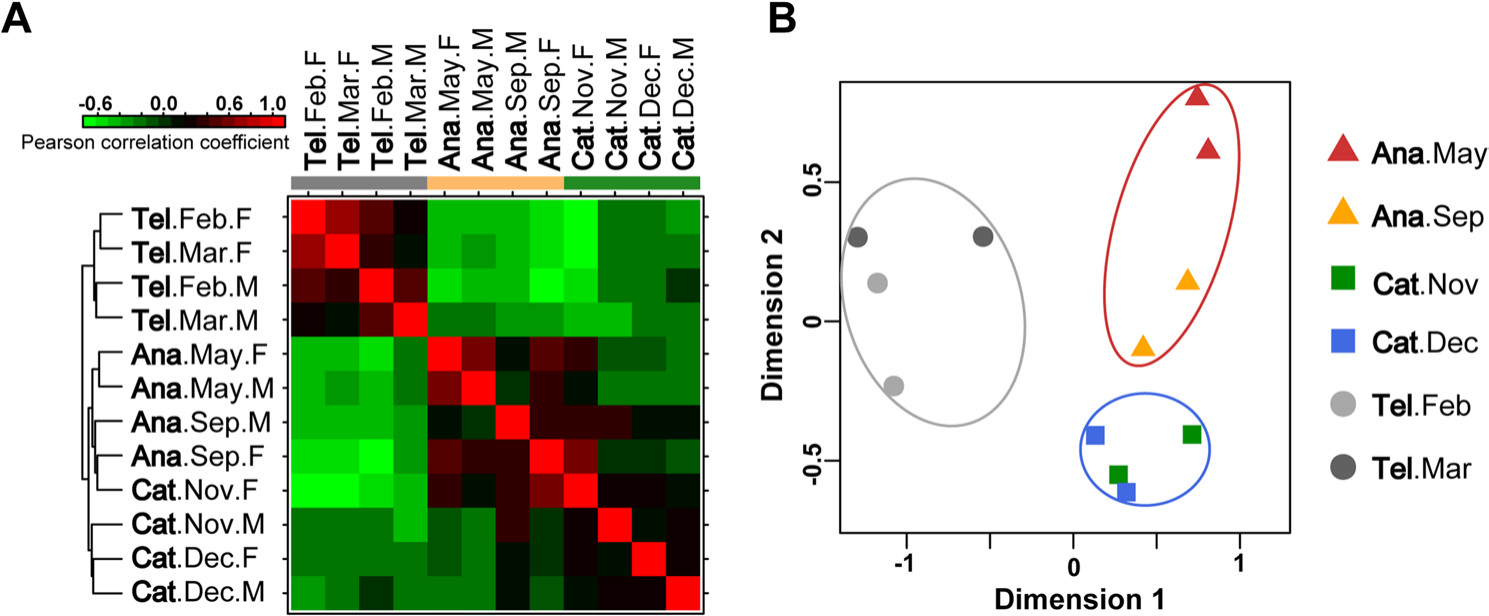
Analysis of gene expression. **A** Genome-wide gene expression profile and hierarchical clustering. **B** MDS of the DEGs during the SHF growth cycle. Tel—telogen; Ana—anagen; Cat—catagen; F—female; M—male

To define the dynamic transcriptional programs associated with SHF growth, we analyzed transcriptomic profiles across the annual cycle using our published dataset [30]. Genome-wide hierarchical clustering revealed three major clusters that correspond directly to the three phases of the hair growth cycle (Fig. 2A). Anagen was clustered closer to catagen than telogen, which formed a clearly distinct cluster. Early anagen in May showed a profile similar to mid-anagen in September, although one November sample clustered with September. Variation among biological replicates likely reflects individual differences, including possible sex-based variation in the transition into regression. As an independent approach, multidimensional scaling (MDS) analysis of differentially expressed genes (DEGs) also discovered three unequivocally separated clusters representing telogen, anagen and catagen (Fig. 2B), which coincides with the results of hierarchical clustering, suggesting that the temporal expression trajectories of DEGs accurately define SHF growth states. These results uncovered the concordant expression changes of molecular signatures that function together to orchestrate the seasonal cashmere growth and shedding.

### Prominent molecular signatures during the transition from telogen to early anagen

To identify factors involved in transitions between successive hair cycle phases, we assayed genes differentially expressed across the full cycle. Given the appreciable expression difference between May and September, each was compared to other phases. Molecular signatures were defined stringently as genes exhibiting at least a 1.5-fold change in expression in a given phase relative to other phases and a corrected *p*-value below 0.05. Following these criteria, we identified 4,659 DEGs, which capture the molecular features of each phase and reveal previously uncharacterized regulators (Supplementary Table S2).

**Fig. 3:**
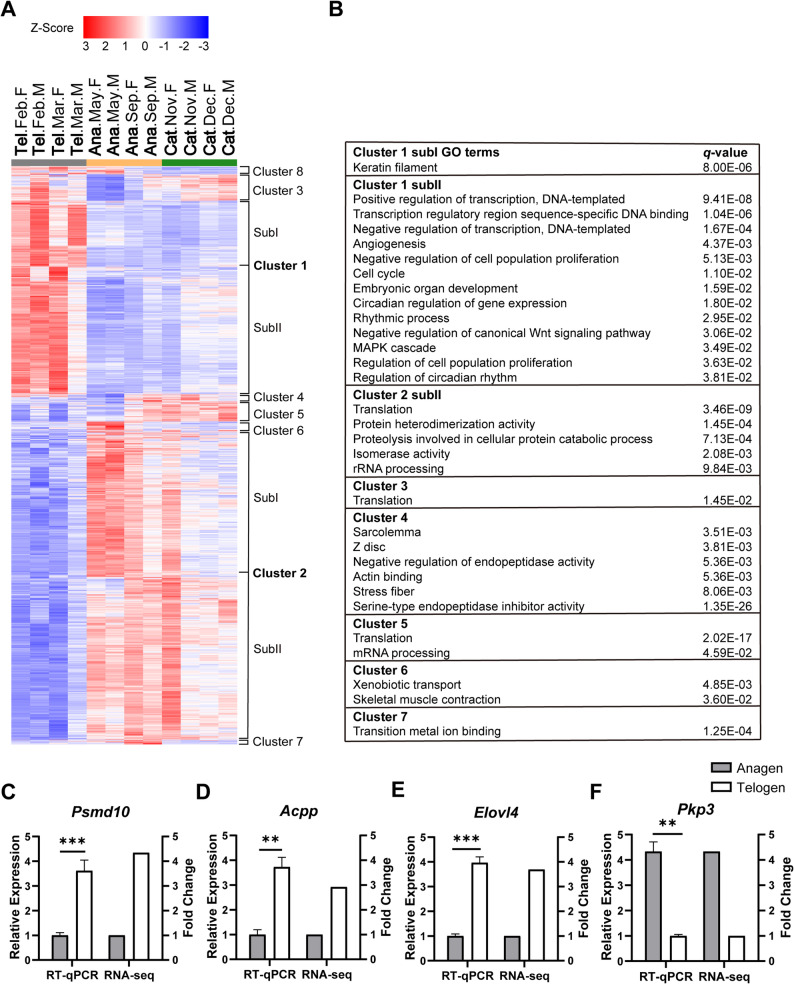
Molecular signatures and expression patterns across the SHF growth cycle. **A** Clustering analysis of the DEGs. Tel—telogen; Ana—anagen; Cat—catagen; F—female; M—male. **B** Enriched GO terms of the DEGs. **C**-**F** Relative expression of *Psmd10* (**C**), *Acpp* (**D**), *Elovl4* (**E**) and *Pkp3* (**F**) in anagen and telogen detected by RT-qPCR (left y-axis). Fold change represents the ratio of mean TPM between telogen and anagen in the sequencing data (right y-axis). *n* = 3 and data are means ± SD for all bar graphs. *P < 0.05, **P < 0.01, ***P < 0.001

Clustering of expression profiles grouped the signature genes into two major and six minor clusters (Fig. 3A), each representing unique expression pattern enriched for significant functional categories (Fig. 3B and Supplementary Table S3). Two predominant clusters, comprising approximately 79.4% of all DEGs, showed sharp expression changes during the transition from telogen to early anagen (May), a pattern distinct from reported miRNA signatures [3]. Therefore, our analysis was primarily focused on this transition. These clusters displayed opposite expression trends, with high expression in telogen (Cluster 1) or in anagen (Cluster 2), suggesting central roles in SHF growth cycling. Both clusters were further divided into two subgroups (subI and subII) according to subtle intra-cluster expression differences.

Subsequent GO analyses showed that signature genes in the telogen phase (Cluster 1) participate in transcription regulation, rhythm regulation, signaling pathways related to Wnt/β-catenin and MAPK, and cell or tissue development (Fig. 3B). Enriched KEGG pathways included circadian rhythm, cellular senescence, Th17/Th1/Th2 cell differentiation, hormone regulation, signaling pathways of MAPK/Wnt/TGF-β/ErbB/FoxO/AMPK/PI3K-Akt and pathways regulating the pluripotency of stem cells (Supplementary Table S3). These findings highlight that telogen is not a dormant state; rather, SHFs engage diverse signaling networks to maintain progenitor cell undifferentiated and prime to initiate a new cycle of hair growth. Notably, genes in the cellular senescence pathway, such as *Tgf-β2*, *Igfbp3* and *E2f-5*, were strongly induced. *Tgf-β2* promotes epithelial apoptosis during regression [33, 34] but also driving proliferation of quiescent hair follicle stem cells (HFSCs) to initiate regeneration [35]. *Igfbp3* antagonizes *Igf-1* and suppresses cell growth [36] and can induce apoptosis independent of IGF signaling [37]. *E2f-5* expression increases during keratinocyte differentiation [38]. We also validated several previously uncharacterized genes in SHFs: *Psmd10*, encoding the oncoprotein Gankyrin, implicated in multiple human cancers through its regulation of autophagy and inflammation [39]; *Acpp*, upregulated in dark skin and implicated in melanosome degradation [40]; and *Elovl4*, whose temperature-induced increase in epidermis accelerates wound healing by shortening inflammation [41] (Fig. 3C-E). Taken together, these identified telogen signatures extend our understanding of previous findings, thereby offering valuable starting points for future work.

Cluster 2 genes were upregulated in anagen. Many declined progressively from catagen (November or December) to their lowest levels in telogen. Functional enrichment indicated roles in translation, protein heterodimerization, isomerase activity, proteolysis, RNA processing, oxidative phosphorylation and thermogenesis (Fig. 3B and Supplementary Table S3). The strong induction of translation-related genes may reflect the overall increased translational activities during anagen. Translation elongation factor *Eef2*, which differs in abundance between guard hair and cashmere in goats [42], whose upregulation may relate to its yet unexplored effects on SHF growth. Proteolysis including several proteases within this cluster may facilitate fiber detachment during exogen [43], consistent with the natural shedding of cashmere in May. The marked increased in *Pkp3* expression (Fig. 3F) is also notable, given that *Pkp3* deficiency impairs hair coat development and increases susceptibility to cutaneous inflammation in vivo [44], underscoring its essential role in maintaining follicle integrity. Our findings indicate that this cluster includes regulators of SHF activity that remain poorly understood.

A smaller cluster (Cluster 6) showed high expression in early anagen (May), followed by a steep decline in mid-anagen. These genes were enriched for extracellular matrix (ECM) receptor interaction, xenobiotic transport, focal adhesion and skeletal muscle contraction (Fig. 3B and Supplementary Table S3). The ECM widely exists in the dermal sheath and dermal papilla during anagen and is essential for hair regeneration [45]. *Col1a2* (collagen type I alpha 2 chain), an ECM-associated protein involved in adhesion and motility, reached the highest expression in the early anagen. Focal adhesions may interact with the ECM to support HFSCs migration toward the bulb [46]. In contrast, Cluster 7 contained genes with higher expression in mid-anagen (September), enriched for transition metal ion binding and the IL-17 signaling (Fig. 3B and Supplementary Table S3).

Signature genes with increased expression in mid-anagen and sustained high expression in catagen (November/December) were clustered into Cluster 4 (decreased from December) and Cluster 5 (decreased from telogen phase). Cluster 4 genes were linked to sarcolemma, Z disc, endopeptidase activity/serine-type endopeptidase inhibitor activity, actin binding and stress fiber organization (Fig. 3B). Cluster 5 genes were functionally related to translation, mRNA processing and oxidative phosphorylation (Fig. 3B and Supplementary Table S3). Interestingly, translation initiation factor *Eif2s2*, present in this cluster, has been implicated in protection against chemotherapy-induced alopecia [47], consistent with its elevated expression in maintaining SHF growth. Two additional clusters corresponded to genes downregulated in early anagen (Cluster 3) or in mid-anagen and catagen (Cluster 8). Mitochondrial-complex-associated gene *Ndufa4* in Cluster 3 is involved in epidermal cell growth and differentiation [48]. Cluster 8 included *Krt1*, *Lor*, and *KLKs* genes, known regulators of keratinocyte differentiation and maturation [49–51]. Numerous DEGs lie outside the major functional categories, suggesting additional biological processes contribute to the regulation of cashmere fiber growth.

### Molecular characterization of keratin and keratin-associated genes in SHFs

**Fig. 4:**
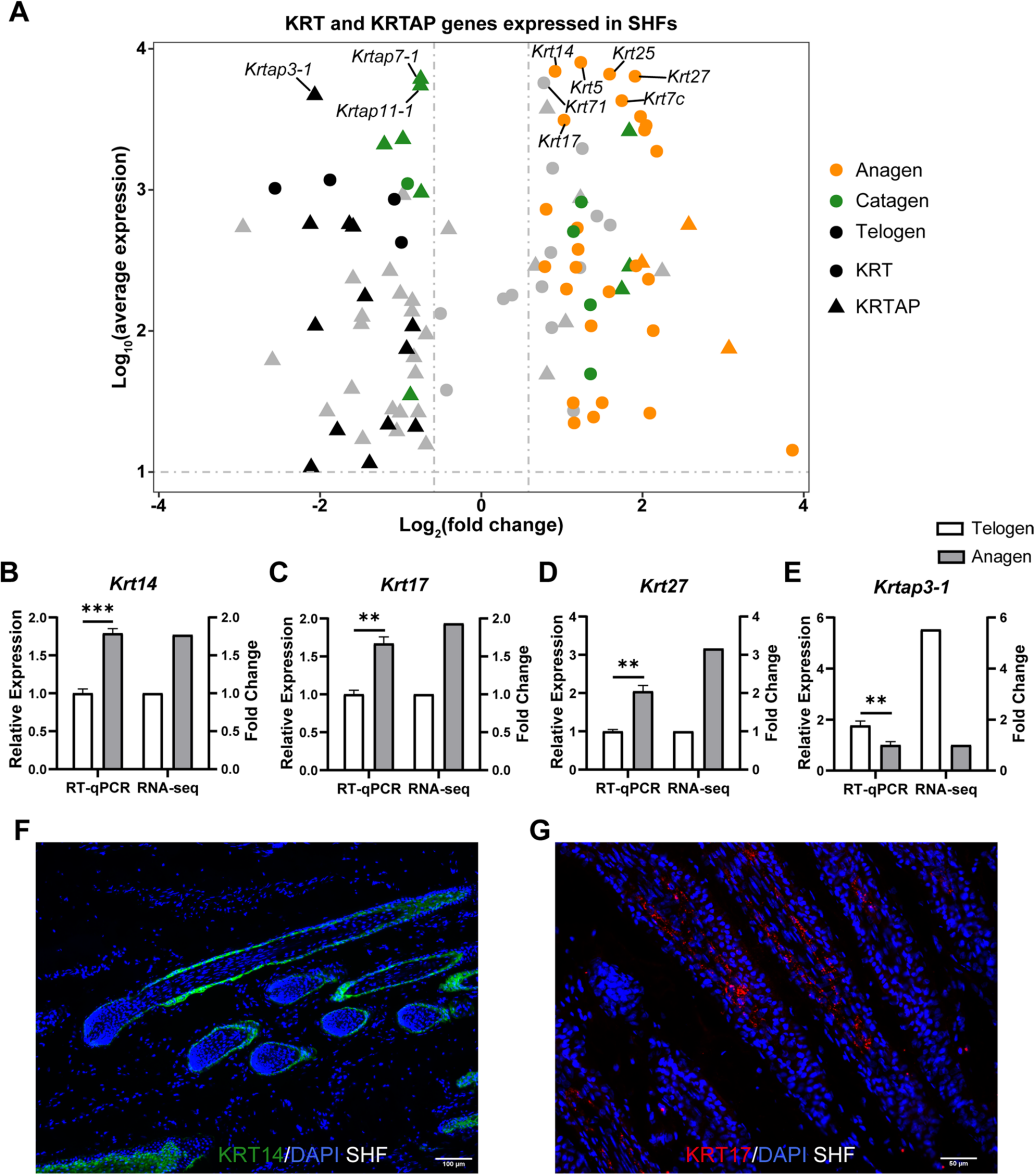
Expression and localization of KRT and KRTAP genes in SHFs. **A** Expression profiles of *KRT* and *KRTAP* genes in SHFs. The y-axis shows the log₁₀-transformed average expression of the phase in which each gene is most highly expressed. The x-axis shows the log₂ fold change of anagen relative to other phases, or catagen relative to telogen. The vertical dotted line marks a 1.5-fold change threshold. Circles and triangles denote *KRT* and *KRTAP* genes, respectively. Colored points indicate DEGs: orange for anagen-upregulated, green for catagen-upregulated, and black for telogen-upregulated. **B**-**E** Relative expression of *Krt14* (**B**), *Krt17* (**C**), *Krt27* (**D**) and *Krtap3-1* (**E**) in anagen and telogen detected by RT-qPCR (left y-axis). Fold change represents the ratio of mean TPM between telogen and anagen in the sequencing data (right y-axis). *n* = 3 and data are means ± SD for all bar graphs. *P < 0.05, **P < 0.01, ***P < 0.001. Significant analysis was performed by one-sided Student’s *t*-test. **F**-**G** Expression of KRT14 (**F**) or KRT17 (**G**) in SHFs was examined by immunofluorescence. The green signals represent KRT14 staining. Scale bars, 100 μm. The red signals represent KRT17 staining. Scale bars, 50 μm. The nuclei were stained with DAPI (blue)

Keratin (KRT) and Keratin-associated proteins (KRTAPs) are major structural components of the hair fiber and its supporting sheath [15, 52]. To investigate their roles during the hair growth cycle, we analyzed their expression patterns in SHFs. Fifty KRT and fifty-three KRTAP genes were expressed at TPM ≥ 10 in at least two samples (Fig. 4A). Among them, 23 KRT and 16 KRTAP genes showed markedly high expression (average TPM > 500), with *Krt5*, *Krt14*, *Krt25*, *Krt27*, *Krt71*, *Krt7c* (*LOC102185436*), *Krtap7-1*, *Krtap11-1* and *Krtap3-1* each exceeding an average TPM of 4,000. The most abundant keratins, *Krt5* and *Krt14*, known epidermal stem cell markers, are typically expressed in basal and suprabasal layer of the outer root sheath (ORS) [53]. By contrast, *Krt25*, *Krt27*, *Krt71*, and *Krt7c* are expressed in the inner root sheath (IRS) and medulla [16, 53]. *Krtap7-1* and *Krtap11-1* showed high expression in the hair matrix and hair fiber cortex, consistent with its established roles in stabilizing the keratin matrix [21, 23].

Across the annual cashmere growth cycle, 36 KRT and 25 KRTAP genes showed phase-specific differential expression. Although *Krt71* was not identified as DEG by statistical test, its expression increased by about 1.5-fold in anagen relative to catagen and telogen. Most expressed KRT genes (about 78%) reached their highest levels in anagen, whereas roughly 12% and 10% peaked in telogen and catagen, respectively. In contrast, a majority of KRTAP genes (about 57%) were most strongly expressed in telogen, with ~ 25% and ~ 18% peaking in catagen and anagen. RT-qPCR validation confirmed these patterns: *Krt14*, *Krt17*, and *Krt27* were expressed at higher levels in anagen than in telogen, whereas *Krtap3-1* was more highly expressed in telogen (Fig. 4B-E).

We next assessed KRT14 and KRT17 localization by immunofluorescence. In the anagen SHFs, high KRT14 activity was restricted to the bulge and ORS (Fig. 4F), while KRT17 signals was confined to the companion layer (Fig. 4G). Together, these findings provide a comprehensive characterization of KRT and KRTAP gene expression in SHFs and support important regulatory roles in annual cashmere fiber growth.

### Co-expression network analysis for SHFs

**Fig. 5:**
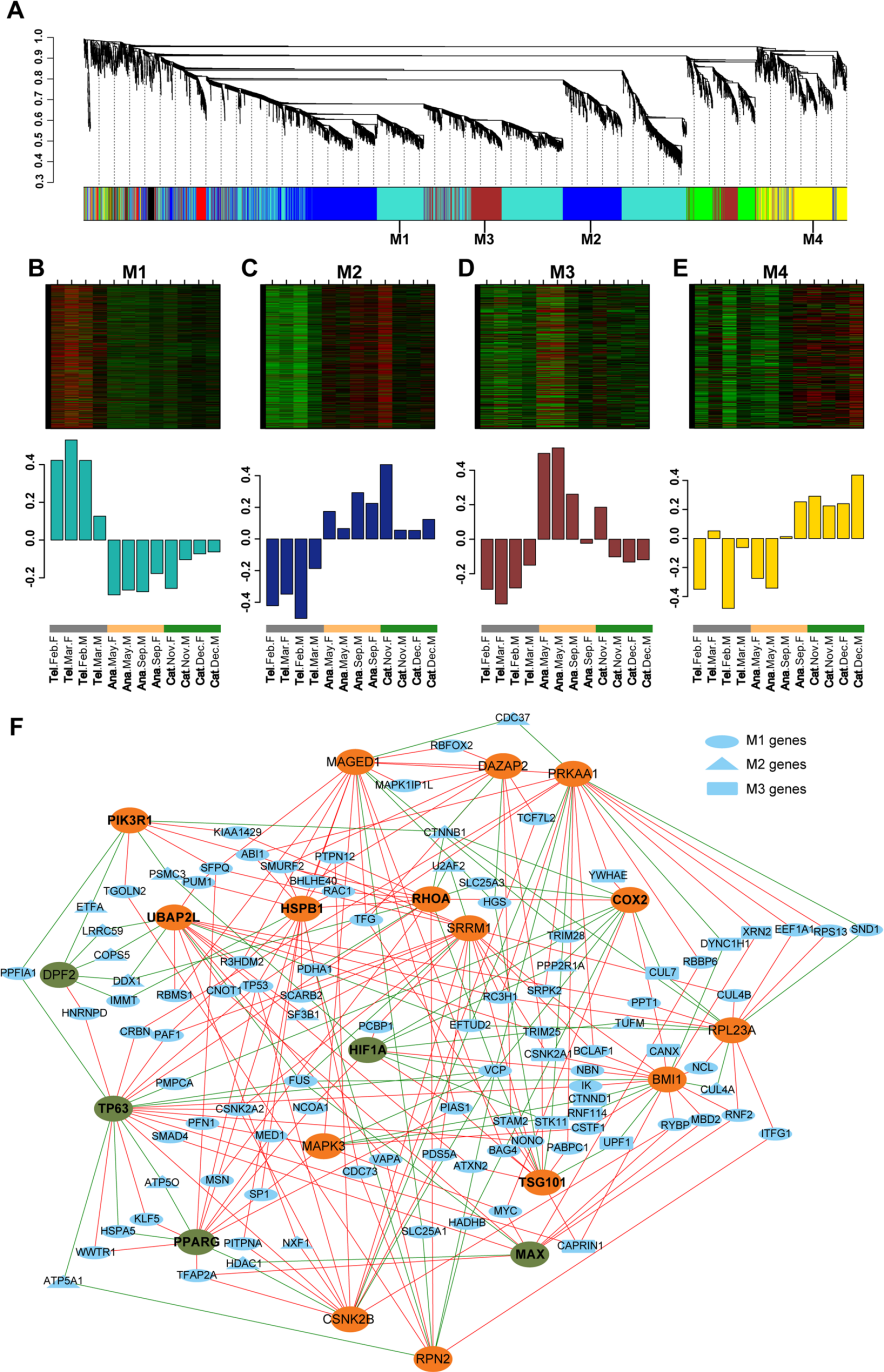
Network analysis of gene expression in SHFs identified distinct modules of co-expressed genes. **A** Dendrograms produced by hierarchical clustering of genes on the basis of topological overlap. Modules of co-expressed genes were assigned colors and numbers as indicated by the horizontal bar beneath each dendrogram; turquoise (M1), blue (M2), brown (M3), yellow (M4). **B**-**E** Modules correspond to different phases during the SHF growth cycle. Heatmaps depicting expression levels for genes of each module: M1 (**B**), M2 (**C**), M3 (**D**), M4 (**E**). Red—increased expression; black—neutral expression; green—decreased expression. (Lower) Barplots of the values of the module eigengene (ME). **F** Network of hub genes from telogen module (M1). The genes that were reported to function in HF/skin growth or development are indicated in bold. The large nodes denote hub genes. The node colors for transcription factor, hub gene and interacting gene are dark green, orange and light blue, respectively. Correlated and anticorrelated interactions are shown as red and green edges, respectively

To study the underlying relationships among genes manifested in the expression profiles at the systematic level, we constructed a weighted gene co-expression network based on pair-wise Pearson correlations for all expressed genes [54]. Genes with high topological overlap were grouped into modules based on unsupervised hierarchical clustering, which identified eight co-expressed gene modules across the annual cashmere growth cycle (Fig. 5A).

Functional relevance of modules was then assessed using heatmaps, which showed that the identified modules correspond closely to the hair growth cycle (Fig. 5B-E). To provide an unbiased basis for module characterization, we performed singular value decomposition and calculated module eigengene, which corresponds to the average of normalized gene expression for a given sample. We focused on the top four modules, which showed significant relationships with specific phases of the hair cycle. The turquoise module (M1) was enriched for genes highly expressed in telogen. The blue module (M2) contained genes with peak expression throughout anagen, followed by reduced expression in catagen. The brown module (M3) comprised genes enriched in early anagen, and the yellow module (M4) consisted of genes most strongly expressed in catagen (Fig. 5B-E).

To identify key regulators of the SHF growth cycle within the largest telogen associated module (M1), we ranked genes in the telogen module by module membership (MM), which reflects the extent of a gene conforms to the characteristic expression pattern of a module. Twenty hub genes were selected for further analysis. Several of these have established functions in hair follicle or skin growth and/or development. For example, deletion of *Pparg* in HFSCs induces a scarring alopecia–like phenotype characterized by hair follicle loss and perifollicular inflammation [55, 56]; *Hif1a* is localized to the inner root sheath and hair shaft and functions as a key regulator of hair regeneration by promoting dermal papilla growth [57–59]; *Maged1*, an outer root sheath-localized protein, is required for normal catagen progression, as its knockout leads to specific catagen defects [60]; *Bmi1* contributes to melanocyte stem cell maintenance, with its loss affecting hair shaft length, while co-overexpression of *Bmi1* and *Tert* enhances the hair-inductive capacity of dermal papilla cells [61–63]; *Tp63* is required for epidermal and follicular renewal [64]; *Max* localizes to the IRS and participates in hair cycle regulation [65]; *Pik3r1* is implicated in atopic dermatitis and shows follicle specific expression [66]; *Hspb1* contributes to stratum corneum formation [67]; *Rhoa* regulates keratinocyte migration [68]; *Tsg101* induces early keratinocyte differentiation [69]; and *Ubap2l* suppresses fibroblast proliferation [70].

To investigate the potential regulatory roles of these twenty hub genes in SHF growth and regression, we constructed a gene interaction network linking hub genes with their correlated or anticorrelated partners (Fig. 5F). Interactions were defined by strong Pearson correlation coefficient (PCC) (|PCC|≥ 0.9). Pathways of two to three nodes connecting pairs of hub genes were retrieved from the BioGRID database (http://thebiogrid.org/, accessed on 25 April 2023) and retained only when supported by strong correlation (Fig. 5F). This integrated network highlights candidate regulatory relationships that may coordinate SHF transitions between telogen and active growth.

## Discussion

By integrating continuous histological observations with transcriptomic profiling of isolated SHFs across an entire year, our study provides a precise delineation of the annual cycle. The combined data indicate that SHFs transition into telogen between February and March, enter anagen from April through October, and undergo catagen between November and January. In the transcriptomic analysis, one November replicate clustered with the September samples. This divergence likely reflects individual heterogeneity and may be influenced by sex-dependent variation in the timing of regression, consistent with our observation of sex-specific differences in molecular signatures. Elucidating the contribution of sex to SHF cycle transitions will require dedicated investigation in future studies.

This study provides the comprehensive, year-long longitudinal morphological analysis of SHFs in Liaoning cashmere goats. Cryosectioning coupled with nuclear fluorescence labeling enabled visualization of the complete follicular longitudinal architecture, allowing direct assessment of hair bulb volume, structural remodeling, and progressive elongation and regression throughout the annual cycle. Previous studies have characterized the annual hair follicle cycle in cashmere goats primarily using transverse histological sections [71, 72], inferring phase transitions based on changes in follicle density and bulb enlargement or shrinkage. In contrast, our longitudinal approach clearly delineates the overall architecture of individual follicles and captures progressive morphological transitions over time. The dermal papilla was clearly resolved, enabling precise comparison of bulb morphology and papilla configuration. Although DAPI staining does not provide the same level of structural detail as hematoxylin and eosin staining for inner and outer root sheaths, it robustly visualizes overall follicular architecture. Samples were collected over nine months, excluding June–August, when SHFs remained in a sustained growth phase without discernible stage-specific morphological changes; histological analyses were therefore not performed during this period. This approach advances captured intermediate morphological states and yielded high-fidelity benchmarks for defining anagen, catagen, and telogen with unprecedented precision.

To investigate transcriptional programs with high accuracy, we performed RNA sequencing on SHFs isolated under microscopy rather than whole-skin samples. This strategy eliminates confounding signals from epidermis, dermal tissue, and primary hair follicles, ensuring that the molecular signatures identified here preferentially expressed in SHFs rather than PHFs or mixed skin tissue. Although some have isolated follicles using enzymatic digestion or mechanical plucking [73–75]. However, these methods may compromise follicular integrity and introduce bias due to incomplete follicle structures, thereby potentially confounding the results. Our genome-wide expression profiling unequivocally distinguished the three phases of the cycle, demonstrating that SHF transcriptomes reliably encode follicular growth states. Moreover, transcriptomic profiling aligned to the morphologically defined stages validated the biological accuracy of the phasic framework, revealing strong concordance between structural dynamics and stage-specific gene expression patterns.

It was notable that the dominant transition occurred from telogen to early anagen. Approximately 79.4% of all differential signatures were associated with the shift from telogen to early anagen, whereas only 13.6% marked the catagen–telogen transition. This imbalance underscores the complexity and regulatory intensity of anagen initiation, during which coordinated gene expression programs prepare the follicle for rapid metabolic activation and fiber synthesis.

Our transcriptomic analyses further revealed that telogen is not a quiescent period, as traditionally described, but rather a transcriptionally active state enriched for Wnt/β-catenin, MAPK, TGF-β, immune-related, and circadian pathways. This finding aligns with observations from murine studies, which report that signatures expressed during telogen are predominantly associated with rhythmic processes, cholesterol metabolism, and activation of canonical Wnt and MAPK signaling pathways [76–79]. The induction of senescence-associated genes—including *Tgf-β2*, *Igfbp3*, and *E2f5*—suggests a sophisticated interplay of apoptotic priming, progenitor-cell maintenance, and tissue homeostasis that poises SHFs for re-entry into anagen. These findings expand the functional definition of telogen and highlight its pivotal role in establishing the regenerative competence of the follicle. Anagen, by contrast, was characterized by strong activation of translational machinery, RNA processing, oxidative phosphorylation, and thermogenic pathways, reflecting the substantial biosynthetic and energetic demands of active fiber production. This pattern is consistent with findings reported in murine studies [76]. Elevated expression of *Eef2*, *Pkp3*, and several proteases points to previously unrecognized mechanisms that coordinate epithelial expansion, fiber assembly, and eventual fiber detachment. In particular, robust induction of *Pkp3*, a stabilizer of epithelial junctions, underscores the importance of adhesion dynamics during rapid follicular growth.

This study provides a systematic comparative analysis of the KRT and KRTAP families across the annual hair follicle cycle, identifying preferentially expressed members in SHFs and clarifying their phase-specific regulatory patterns. The contrasting temporal expression profiles of KRT and KRTAP families across phases provide new insights into the molecular coordination of fiber formation. Most *KRT* genes peaked in anagen, reflecting their role in assembling the cortical and medullary framework of the hair shaft. In contrast, *KRTAP* genes were predominantly upregulated in telogen and catagen, a pattern that differs from previous observations in whole-skin transcriptomic analysis [72]. This inverse expression timing suggests that cortex-forming keratins and cuticle-associated KRTAPs are under distinct regulatory controls, with potential consequences for fiber diameter, fineness, and tensile properties.

## Conclusions

Together, these results establish a biologically validated, high-resolution phase framework for the cashmere growth cycle, grounded in morphology and matched transcriptomic signatures. By integrating structural dynamics with molecular regulation across an entire year, these results establish a foundational resource for the field, advance mechanistic insight into synchronized follicle renewal, and provide molecular targets with potential relevance for improving cashmere fiber production and for broader applications in hair biology and regenerative dermatology.

## Supplementary Information

Supplementary Material 1: Table S1. Primer sequences for mRNA RT-qPCR.

Supplementary Material 2: Table S2. List of differentially expressed genes.

Supplementary Material 3: Table S3. Enriched KEGG pathway analysis of the differentially expressed genes.

## Data Availability

The raw RNA-seq data and gene expression values have been deposited in the NCBI Gene Expression Omnibus (GEO) database under accession number GSE221472. The detailed information on the data processing pipeline has been described in our previous study [30]. The list of differentially expressed genes (DEGs) identified in this study is provided in Supplementary Table S2.

## Abbreviations

HF: Hair follicle
SHF: Secondary hair follicle
PHF: Primary hair follicle
DEG: Differentially expressed gene
KRT: Keratin
KRTAP: Keratin-associated protein
