# Supplementary material for: Molecular characterization and functional insights of secondary hair follicles across the cashmere growth cycle
Source: BMC Genomics. 2026 Apr 25;27:539. doi: 10.1186/s12864-026-12885-7 (PMC13262452; doi:10.1186/s12864-026-12885-7)
Supplement: Supplementary file 1 — Supplementary Material 1: Table S1. Primer sequences for mRNA RT-qPCR. [file 12864_2026_12885_MOESM1_ESM.docx]

| **Name** | **Forward primer sequence (5′ to 3′)** | **Reverse primer sequence (5′ to 3′)** |
| --- | --- | --- |
| *Psmd10* | CCAAGGAGCAAGCATTTA | GTATCAAACCCAGCCCAC |
| *Acpp* | CCAGAGCAACAAGCCTTCA | TCGGTCTCCATGCCTAAAA |
| *Elovl4* | GTCTCCACTGCCTACACTTT | AGGTTCTCGGTCCTTCATC |
| *Pkp3* | TGAGGATAAGAGCGTGGAGA | TGAAGGGCTGAGGGTGG |
| *Krt14* | GCGTGGGTAGTGGTTTTGGT | CAGGCGGTCATTCAGGTTC |
| *Krt17* | ACCACCATCCGCCACTT | AGCCACCGCCGACAAA |
| *Krt27* | ACCTACTGCCGCCTGAT | GTTGACTTTGGTGGACTTCT |
| *Krtap3-1* | TGCTCAACTCTTCCCACC | GGCTCGCAGGCATTTT |
| *Actb* | GGCAGGTCATCACCATCGG | CGTGTTGGCGTAGAGGTCTTT |

**Supplementary Material 1: Table S1**

**Table S1. Primer sequences for mRNA RT-qPCR**
