# Supplementary material for: Molecular characterization and functional insights of secondary hair follicles across the cashmere growth cycle
Source: BMC Genomics. 2026 Apr 25;27:539. doi: 10.1186/s12864-026-12885-7 (PMC13262452; doi:10.1186/s12864-026-12885-7)
Supplement: Supplementary file 3 — Supplementary Material 3: Table S3. Enriched KEGG pathway analysis of the differentially expressed genes. [file 12864_2026_12885_MOESM3_ESM.docx]

**Supplementary Material 3: Table S3**

**Table S3. Enriched KEGG pathway analysis of the differentially expressed genes**

| **Cluster 1 subI KEGG pathways** | ***q*-value** |
| --- | --- |
| Herpes simplex virus 1 infection | 3.83E-02 |
| **Cluster 1 subII** |  |
| Th17 cell differentiation | 1.55E-03 |
| MAPK signaling pathway | 2.93E-03 |
| Cellular senescence | 2.93E-03 |
| Parathyroid hormone synthesis, secretion and action | 2.93E-03 |
| FoxO signaling pathway | 2.93E-03 |
| Circadian rhythm | 2.93E-03 |
| Signaling pathways regulating pluripotency of stem cells | 4.24E-03 |
| Wnt signaling pathway | 4.59E-03 |
| TGF-beta signaling pathway | 5.33E-03 |
| ErbB signaling pathway | 5.35E-03 |
| Thyroid hormone synthesis | 9.84E-03 |
| Th1 and Th2 cell differentiation | 1.21E-02 |
| Relaxin signaling pathway | 2.00E-02 |
| AMPK signaling pathway | 2.66E-02 |
| PI3K-Akt signaling pathway | 2.66E-02 |
| **Cluster 2 subII** |  |
| Oxidative phosphorylation | 8.32E-14 |
| Thermogenesis | 2.77E-11 |
| **Cluster 3** |  |
| Oxidative phosphorylation | 2.10E-02 |
| **Cluster 5** |  |
| Oxidative phosphorylation | 2.76E-03 |
| **Cluster 6** |  |
| ECM-receptor interaction | 1.69E-02 |
| Focal adhesion | 1.74E-02 |
| **Cluster 7** |  |
| IL-17 signaling pathway | 1.42E-07 |
